# Electrochemical Impedance Immunosensor Based on Self-Assembled Monolayers for Rapid Detection of *Escherichia coli* O157:H7 with Signal Amplification Using Lectin

**DOI:** 10.3390/s150819212

**Published:** 2015-08-05

**Authors:** Zhanming Li, Yingchun Fu, Weihuan Fang, Yanbin Li

**Affiliations:** 1 College of Biosystems Engineering and Food Science, Zhejiang University, Hangzhou 310058, China; E-Mails: lizhanming1018@126.com (Z.L.); yingchunfu@126.com (Y.F.); 2 College of Animal Science, Zhejiang University, Hangzhou 310058, China; E-Mail: whfang@zju.deu.cn; 3 Department of Biological & Agricultural Engineering, University of Arkansas, Fayetteville, AR 72701, USA

**Keywords:** *Escherichia coli* O157:H7, impedance immunosensor, lectin, screen-printed interdigitated microelectrode

## Abstract

*Escherichia coli* O157:H7 is a predominant foodborne pathogen with severe pathogenicity, leading to increasing attention given to rapid and sensitive detection. Herein, we propose an impedance biosensor using new kinds of screen-printed interdigitated microelectrodes (SPIMs) and wheat germ agglutinin (WGA) for signal amplification to detect *E. coli* O157:H7 with high sensitivity and time-efficiency. The SPIMs integrate the high sensitivity and short response time of the interdigitated electrodes and the low cost of the screen-printed electrodes. Self-assembling of bi-functional 3-dithiobis-(sulfosuccinimidyl-propionate) (DTSP) on the SPIMs was investigated and was proved to be able to improve adsorption quantity and stability of biomaterials. WGA was further adopted to enhance the signal taking advantage of the abundant lectin-binding sites on the bacteria surface. The immunosensor exhibited a detection limit of 10^2^ cfu·mL^−1^, with a linear detection range from 10^2^ to 10^7^ cfu·mL^−1^ (*r*^2^ = 0.98). The total detection time was less than 1 h, showing its comparable sensitivity and rapid response. Furthermore, the low cost of one SPIM significantly reduced the detection cost of the biosensor. The biosensor may have great promise in food safety analysis and lead to a portable biosensing system for routine monitoring of foodborne pathogens.

## 1. Introduction

*Escherichia coli* O157:H7, a common member of a group of pathogenic *E. coli* strains, has been marked as an enterohaemorrhagic, verocytotoxin-producing, or Shiga-toxin-producing pathogen [1]. In addition to devastating personal losses to the patients, the economic costs can be substantial—the costs of healthcare, social care, and lost productivity come to around $600 million per year in the United States, whereas costs from product recalls and reduced trade can run to tens of millions of dollars [2,3]. *E. coli* O157:H7 is the predominant serotype associated with human health and food safety [4,5]. Human infections are usually caused by the consumption of contaminated and under-cooked beef, unpasteurized milk, and feces-contaminated vegetables and water [6,7]. Deaths are usually associated with severe extra-renal complications [1]. Considering the serious threats from *E. coli* O157:H7, it is of great importance to develop rapid and sensitive detection methods to ensure food safety. The early detection at low concentrations still remains a challenge to allow immediate decisions to be made in many important fields.

There are many detection methods for *E. coli* O157:H7, such as traditional culture methods, immuno-assays, and molecular methods. Although these methods have high sensitivity and reliability, their application to food safety is limited due to time-consuming and complicated operations [8]. Studies on biosensing methods have increased significantly in the recent years, and become one of the most active research areas for pathogen detection [9,10]. Numerous biosensors have been developed for detection and enumeration of *E. coli* O157:H7, and some of them are promising candidates for rapid screening [11,12,13]. Electrochemical biosensors are widely recognized as powerful analytical tools due to a variety of contributions such as simple instrumentation, easy operation, low cost and short response time [14,15]. Some impedimetric biosensors based on different types of electrodes have recently been reported for pathogen detection, and disposable screen-printed electrodes offer cost-effective methods [16,17]. The increasing commercial availability of low-cost screen-printed electrodes has opened new fields for electrochemical measurements [18]. Interdigitated microelectrodes can maximize the impedance change, reduce the detection time, minimize interfering effects, and work in a two-electrode system (*vs.* conventional three-electrode system), thus significantly benefiting the fabrication and performance of electrochemical biosensors [19,20,21]. A combination of two electrodes, namely, screen-printed interdigitated microelectrodes (SPIMs), may integrate their merits to develop highly sensitive, rapid-responding, cost-effective biosensors for the detection of *E. coli* O157:H7. However, related research is rarely reported.

Self-assembled monolayers (SAMs) are broadly used to functionalize the electrode surface, improve adsorption capacity and stability, and retain activity of the biomaterials [22,23,24]. Covalent binding (amide bond formation) results from the reaction between amino group of protein and reactive succinimidyl group of the 3-dithiobis-(sulfosuccinimidylpropionate) (DTSP) on the SAM surface. DTSP can be easily used without strict reaction conditions and additional activation step, compared with other SAMs, such as 4,4-Dithiodibutyric acid (DTBA) and 3-Mercaptopropionic acid (MPA). DTBA and MPA SAMs need to activate with N-Hydroxysuccinimide (NHS)/carbodiimide hydrochloride (EDC) before use under strict reaction conditions which increase the complexity [25,26,27]. The short self-assembling time and mild reaction conditions improve the accuracy and practicality of the microelectrode surface functionalization, which is useful for modification of biomaterials.

More recently, lectin binding sites at the surface of a high percentage of microorganisms can be used for bacteria absorption, which has proven to be greatly promising and effective. Lectins are much smaller than antibodies, thus they allow higher absorption capacity leading to higher sensitivity and lower non-specific adsorption. Furthermore, lectins are readily available and inexpensive recognition agents [28,29,30]. As far as we know, the application of wheat germ agglutinin (WGA) as signal amplification for bacteria detection has never been reported.

**Scheme 1:**
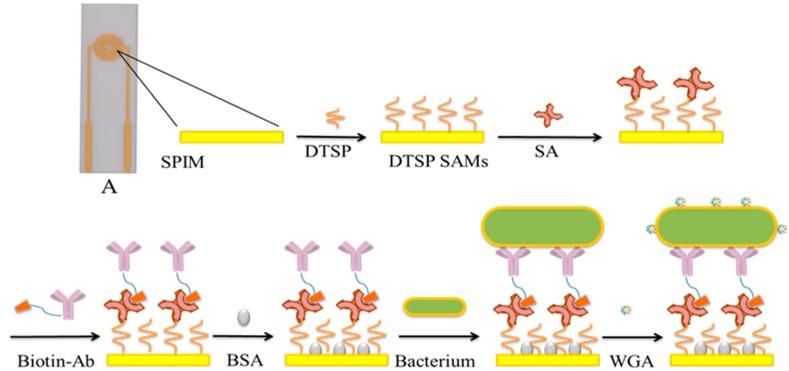
A scheme of the proposed immunosensor for bacteria detection. A was a photo of SPIM and the width of a finger and the gap between two fingers of this microelectrode were 200 μm, respectively.

Herein, an electrochemical impedance immunosensor based on self-assembled monolayers was proposed for rapid detection of *E. coli* O157:H7 with signal amplification using WGA (Scheme 1). With the immobilization of the biomaterials onto the surface of the SPIMs, the impedance signal can be changed due to the blocking of electron transfer, which can be utilized as the sensing mechanism of impedance immunosensor. Compared with the expensive non-screen printed microelectrodes, the cost of one SPIM may be less than $1 which is much better for the practical application and can be further reduced by the good reusability. The performance of the electrochemical impedance biosensor showed that this biosensor was sensitive, reliable and effective for detection of *E. coli* O157:H7, which hold great promise in food safety analysis.

## 2. Experimental Section

### 2.1. Apparatus and Reagents

Electrochemical impedance measurements were performed using the ZAHNER chemical station (Kronach, Germany). SPIMs were purchased from AIBIT Biotech Instrument (Jiangyin, China). The width of a finger and the gap between two fingers for the gold interdigitated microelectrode were 200 μm, respectively. Gold interdigitated microelectrodes with 25 μm width and gap were obtained from Institute of Semiconductors of Chinese Academy of Sciences (Beijing, China). Scanning electron microscopy (Hitachi, Japan) was used for the characterization of electrodes surface.

DTSP, MPA, WGA, N-Ethyl-N'-(3-dimethylaminopropyl) carbodiimide hydrochloride (EDC), N-Hydroxysuccinimide (NHS) and phosphate buffer solutions (PBS, pH 7.4) were purchased from Sigma (St. Louis, MO, USA). *E. coli* K12 (ATCC29425), *E. coli* O157:H7 (ATCC43889), *Listeria monocytogenes* (ATCC19151) and *Salmonella* Typhimurium (ATCC14028) were purchased from ATCC (Manassas, MD). Streptavidin (SA), bovine serum albumin (BSA) and protein A were purchased from Sangon Biotech (Shanghai, China). MacConkey agar, brain heart infusion (BHI) culture medium (Becton, Dickinson and Company, Sparks, NV), and nutrient agar (Huankai Microbial Sci. & Tech. Co. LTD, Guangzhou, China) were used for the bacteria culture. Anti-*E. coli* biotin-antibodies (biotin-Ab) were obtained from Meridian Life Science (Saco, ME) and dissolved in PBS (0.4 mg·mL^−1^). DTSP was dissolved in acetone (2 mM). PBS solution containing 10 mM K_3_Fe(CN)_6_/K_4_Fe(CN)_6_ (Sangon Biotech., Shanghai, China) was used for electrochemical impedance spectroscopy (EIS) measurements. Ultrapure water (18.2 MΩ·cm) was obtained from a Millipore Milli-Q purification system (Merck Millipore, MA). All chemicals were of analytical grade. All the bacteria dilutions used in this research were inactivated (10 min in boiling water).

### 2.2. Preparation of Bacterial Samples

*E. coli* O157:H7 was grown in BHI culture medium at 37 °C for 20 h to the stationary phase. Serial 10-fold dilutions of the culture were made with PBS and 100 μL of the diluted solutions were transferred to MacConkey agar plates and incubated for 24 h at 37 °C for enumeration of colonies. At the same time, the stationary-phase cultures were diluted to 10^1^–10^7^ cfu·mL^−1^ in PBS (pH 7.4) for use in the tests. *S.* Typhimurium and *E. coli* K12 were also incubated at 37 °C for 24 h and *L. monocytogenes* was incubated for 48 h in BHI culture medium. Incubated microorganisms were serially diluted in PBS and bacterial numbers were determined using a conventional plate counting method. The dilutions containing approximately 10^5^ cfu·mL^−1^ of each microorganism were prepared for evaluation of the specificity of the proposed impedance immunosensor.

### 2.3. Immunosensor Fabrication

Two methods were adopted to clean the bare SPIMs. For the first one, NaOH solution (50 μL, 1 M) was dropped on the electrode area, kept for 5 min, and washed with water, followed by similar treatment of HCl solution (50 μL, 1 M) for 2 min. For the second method, piranha solution (H_2_SO_4_:H_2_O_2_ (v/v) = 7:3, 50 μL) was dropped onto the electrode surface and kept for 15 min, followed by washing with water thoroughly. After the cleaning procedure, the microelectrodes with different sizes (25 µm and 200 µm width) were characterized using cyclic voltammetry (CV) and EIS.

Self-assembly was carried out by immersing electrodes in 2 mM DTSP. The effect of different incubation times (0, 2, 4, 8 h) on sensor performance was studied. After incubation, the microelectrodes were washed immediately using acetone to remove free DTSP. The self-assembled monolayers of MPA were obtained and activated with EDC + NHS before use [31]. Briefly, EIS was used to characterize the electrodes modified with different SAMs.

After DTSP immobilization, the electrodes were incubated in SA solution (50 μL, 0.5 mg·mL^−1^) for 45 min and then washed with ultrapure water. Then electrodes were incubated with biotin-Ab solution for another 45 min and washed with ultrapure water again. Different biotin-Ab concentrations (0.1, 0.2 and 0.4 mg·mL^−1^) were evaluated for optimization. Finally, BSA solution (10 mg·mL^−1^) was applied to block non-specific adsorption sites on the surface of the microelectrodes.

### 2.4. Bacteria Detection

Different dilutions of inactivated bacterial cultures (from 10^1^ to 10^7^ cfu·mL^−1^) were dropped on the surface of electrodes and incubated for 45 min. Impedance measurements were conducted using a 10 mM K_3_Fe(CN)_6_/K_4_Fe(CN)_6_ (1:1) mixture in PBS (1 Hz–1 MHz, 10 mV). Each detection was repeated more than three times. Several inactivated non-target bacteria, including *E. coli* K12, *L. monocytogenes* and *S*. Typhimurium were used as non-target bacteria to evaluate the specificity of this immunosensor.

### 2.5. Lectin Absorption

The WGA solution (50 μL, 0.5 mg·mL^−1^) was used to amplify the impedance signal. The incubation time was 45 min and then washed with ultrapure water. Impedance measurements were also conducted using a 10 mM K_3_Fe(CN)_6_/K_4_Fe(CN)_6_ (1:1) mixture in PBS (1 Hz–1 MHz, 10 mV).

### 2.6. Regeneration of SPIMs

After each test, the used SPIMs were treated with piranha solution to disassociate the bacterial cells and other materials attached on the surface of the SPIMs. Then, impedance data were collected to compare with the initial value. The electrodes were reused if their impedance values were similar to initial values.

## 3. Results and Discussion

### 3.1. Characterization of the Microelectrodes

**Figure 1:**
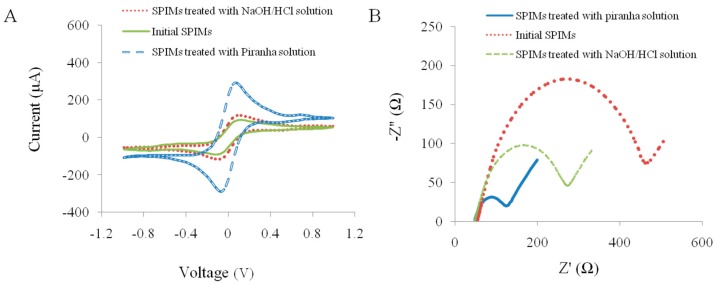
Characterization of the SPIMs by cyclic voltammetry (CV) and electrochemical impedance spectroscopy (EIS) after the washing with piranha solution and NaOH/HCl solution. (**A**) the current change; and (**B**) the impedance change.

To effectively clean these new kind of SPIMs, different cleaning procedures were conducted and compared. CV and EIS methods using a K_3_Fe(CN)_6_/K_4_Fe(CN)_6_ probe were adopted to investigate the cleaning efficiency by comparing peak current of electrochemical probes and electron transfer resistance (*R*_et_), respectively (Figure 1) [31,32]. After being treated with conventional NaOH/HCl and piranha solutions, SPIMs showed the increases of peak currents of the electrochemical probe by 25% and 180%, and the decreases of *R*_et_ by 45% and 75%, respectively, indicating the cleanliness was improved. Obviously, piranha solution was more efficient in cleaning, which was, therefore, selected for further use. We also found that peak current and *R*_et_ kept constant after a 15 min immersion. Accordingly, we could readily obtain a clean SPIM through immerging electrodes into piranha solution for 15 min. This treatment is much more convenient and time-saving compared with treatments commonly used for rod electrodes or plate electrodes, which generally need complicated polishing procedures [33,34,35]. These merits should highlight the convenience of SPIMs and benefit the facile fabrication of biosensors.

**Figure 2:**
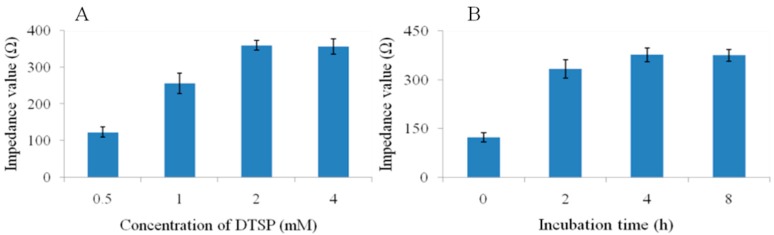
The effect of (**A**) the concentration of DTSP and (**B**) incubation time of DTSP on the impedance value.

The self-assembling of DTSP was monitored through measuring the impedance value at 100 Hz, as shown in Figure 2. After 2 h incubation, impedance values had minor change (*p* > 0.05), indicating nearly full self-assembling, though the arrangement of DTSP molecules might be still going to finally form a perfect monolayer. Here, we chose 4 h incubation time considering both the self-assembling efficiency and time efficiency. Similarly, the concentration of DTSP was also investigated and optimized to be 2 mM.

**Figure 3:**
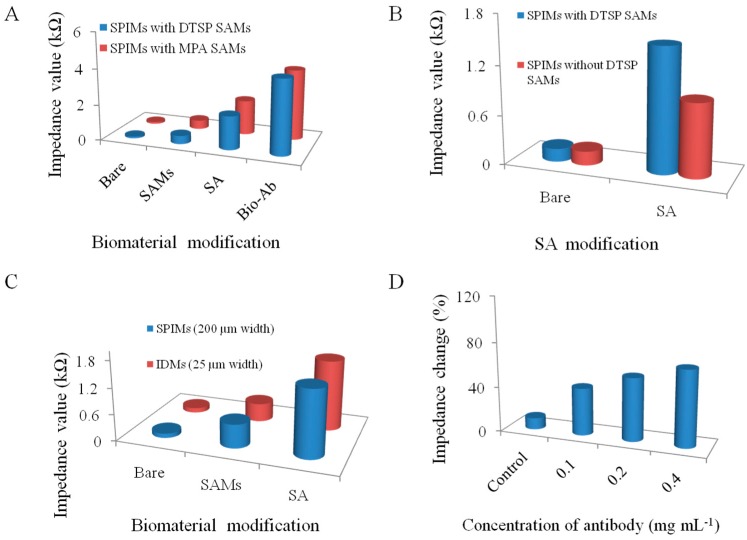
(**A**) The performance of the different SAMs (DTSP SAMs and 3-Mercaptopropionic acid (MPA) SAMs); (**B**) The absorption capacity of SPIMs with or without SAMs; (**C**) Comparison of the SPIMs (200 µm width of electrode fingers) and interdigitated microelectrodes (IDMs, 25 µm width of electrode fingers); and (**D**) The optimization of the antibody concentration. All the experiments were repeated more than three times.

For comparison, a conventional method for the modification of materials on the surface of the SPIMs using MPA SAMs plus EDC/NHS cross-linking was also used to immobilize antibodies (Figure 3A). We found that the immobilization efficiency of the new DTSP-based method performed even better than this classic method. Additionally, the conventional method requires 4 h for self-assembling of MPA plus another 3 h activation of carboxyl group using EDC/NHS. Therefore, DTSP self-assembling should be a better choice with comparable performance but shorter preparation time. Moreover, we evaluated the absorption capacity of SPIMs with or without the DTSP SAMs (Figure 3B) and found that the absorption capacity increased significantly when the SPIM was modified with SAMs. The increase improved detection performance, verifying the necessity of the SAMs.

The impedance changes of the microelectrodes with different sizes are shown in Figure 3C. The results indicated that there was no significant change between the 25 µm and 200 µm microelectrodes (*p* > 0.05) after the biomaterials immobilization. Compared with 25 µm interdigitated microelectrodes, the application of SPIMs is promising due to their low cost.

**Figure 4:**
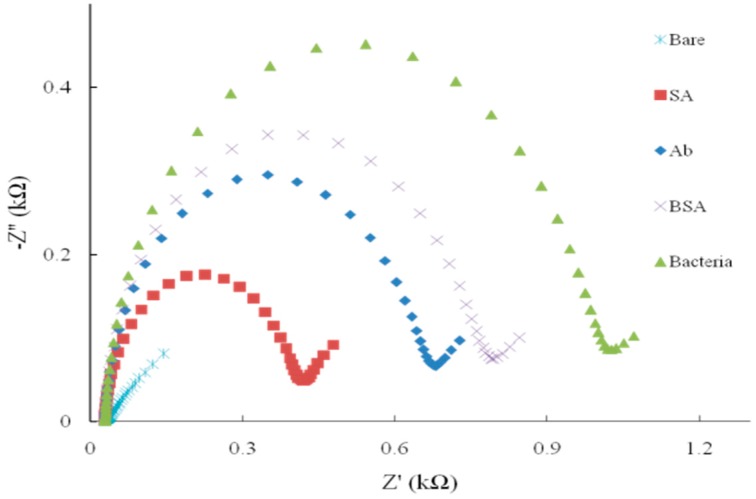
The impedance change after the SPIMs modification with biomaterials (SA, Ab and BSA) and the capture of target bacteria *E. coli* O157:H7 (Nyquist plot).

Besides the self-assembling time and the concentration of DTSP, we also optimized the concentration of the antibody (Figure 3D). The impedance change showed that the adsorbing capacity was induced with the different dilutions. Compared with the control, the lower dilution (0.2 mg·mL^−1^) of antibody was enough for detection of bacteria. There was no significant difference between the concentrations of 0.2 and 0.4 mg·mL^−1^ (*p* > 0.05). The disadvantage of the lower concentration of antibodies is that the sensitivity of the method may be affected due to less amount of the antibody available to capture the target pathogen. Each step of the surface modification was measured and the result was shown in Figure 4. The bare microelectrode showed a very small impedance value (curve a) because of the low resistance of the [Fe(CN)_6_]^3−/4−^ charge transfer process. When SA (curve b), antibody (curve c) and BSA (curve d) were immobilized onto the microelectrode surface, impedance increased, which confirmed the successful surface immobilization.

### 3.2. Detection and Signal Amplification

**Figure 5:**
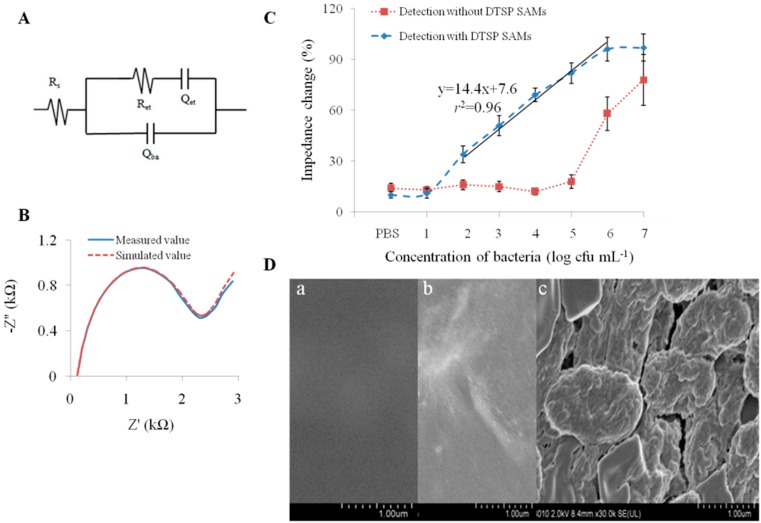
(**A**) The plot of the measured and simulated data using the immunosensor with DTSP SAMs; (**B**) A modified Randles’ equivalent circuit; (**C**) The detection of *E. coli* O157:H7 at different concentrations using the immunosensor with DTSP SAMs or not; (**D**) Characterization of the surface of the bare SPIM (**a**), the surface of the SPIMs before (**b**) and after (**c**) the bacteria incubation by scanning electron microscope.

For better understanding of the detection mechanism, a modified Randles’ equivalent circuit obtained using Zpswin 3.10 software was used to adequately fit the measurement data over the whole frequency range (Figure 5). Wherein, *R*_s_ stands for the resistance of the electrolyte solution and *R_et_* for charge transfer resistance, *Q*_et_ is the constant phase elements associated with the capacitance of the double layer and *Q*_ba_ is the capacitance of the biomaterial absorption. All the impedance spectra of different concentrations of bacteria were fitted to the equivalent circuit. During the modification and detection, the loading of non-conductive proteins and bacteria would significantly retard the interfacial electron-transfer kinetics and increase the electron-transfer resistance, leading to the changes of *R*_et_ values. The *R*_et_ value is the most important electrical parameter in analyzing the impedance signal change for detection of bacteria and can be used to evaluate the detection performance [19,20,21]. When bacterial cells were introduced to the fabricated immunosensor, there was a further increase in *R*_et_, indicating that the microelectrode surface had been attached with a large number of bacterial cells.

**Figure 6:**
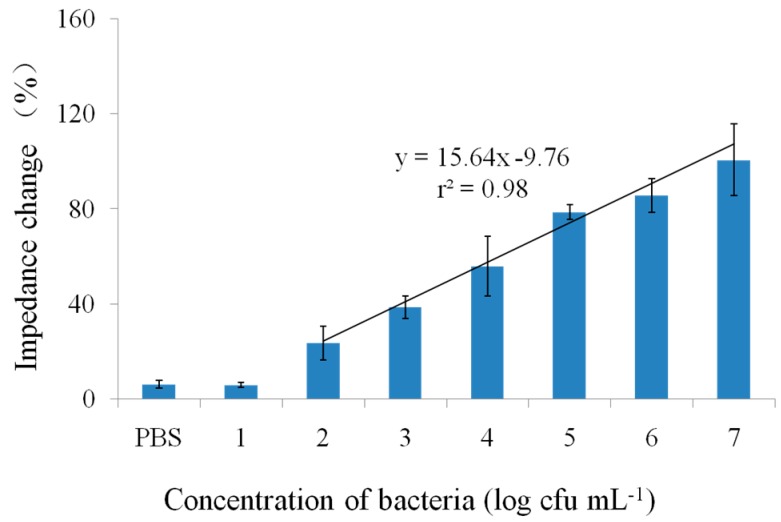
The signal amplification of the immunosensor with WGA solution.

Under optimized conditions, the immunosensor provided a detection limit of 10^2^ cfu·mL^−1^ and a linear detection range (*r^2^* = 0.96) for *E. coli* O157:H7 between 10^2^ and 10^6^ cfu·mL^−1^ (Figure 5C). The total detection time including incubation was less than 1h. The detection performance was comparable to the previous research and the immunosensor was easily fabricated [36,37]. The detection performance of the proposed immunosensor was comparable or better than some of reported biosensors that used interdigitated microelectrodes [19,20,21]. The proposed biosensor presented a lower detection limit and higher specificity, as compared with those of the previously reported biosensors using lectin as recognition element [38,39]. We also evaluated the detection performance of the physical absorption of SA in which only 10^6^ cfu·mL^−1^ and higher concentrations of bacterial cells could be distinguished (Figure 5C). The reasonable explanation was that DTSP SAMs could be used to improve the stability of the immobilization and increase the absorption capacity. Moreover, the bacteria attached onto the surface of the SPIM was characterized by scanning electron microscopy (Figure 5D) demonstrating the good performance of the constructed immunosensor. The absorption of bacteria cells and WGA has been verified in other research [29,30]. WGA molecules can be successfully absorbed by the fabricated biosensor after the bacteria absorption to improve the impedance change compared with control. Compared to the detection with antibody only (blue curve in Figure 5C), the impedance value was enhanced and the value of the *r^2^* was improved from 0.96 to 0.98 (Figure 6). The results demonstrate proof of this concept that lectins, including WGA, can be utilized for signal amplification. Further work can be done to select one lectin with better specificity for the bacteria from different lectins. We have prepared five lectins, including WGA, canavailiaensiformis (Con A), ulexeuropaeus (UEA), M. amurensis (MAL) and arachishypogaea (PNA) for the optimization. Detection parameters’ optimization is also further research for good performance.

**Figure 7:**
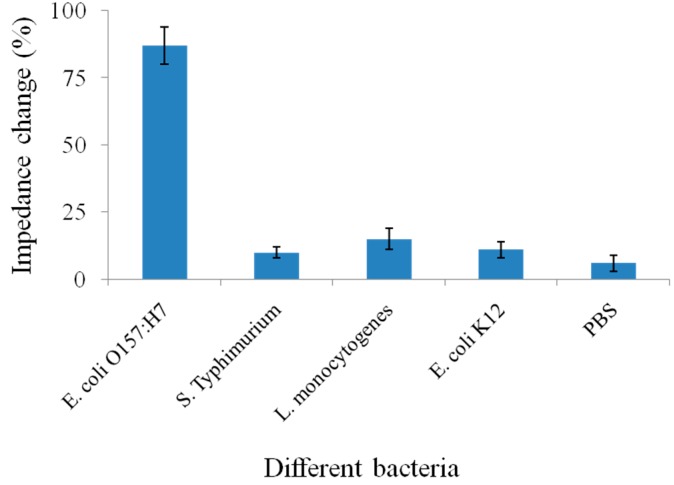
The specificity of the immunosensor for detection of *E. coli* O157:H7.

The specificity of the proposed biosensor was evaluated using *E. coli* K12, *L. monocytogenes* and *S.* Typhimurium as non-target bacteria. There was no significant attachment when these bacteria were incubated, which clearly evidenced the sensing specificity to *E. coli* O157:H7 (Figure 7). Moreover, the cost of one SPIM may be less than $1 which is much better for the practical application compared with the expensive non-screen printed microelectrodes. Moreover, the cost can be further reduced by good reusability. The proposed biosensor has the potential in the development of a simple, low cost and portable biosensing system for rapid and sensitive detection of pathogens other than *E. coli* O157:H7.

## 4. Conclusions

An electrochemical impedance immunosensor based on self-assembled monolayers was proposed for rapid detection of *E. coli* O157:H7 with signal amplification using WGA. The application of WGA as signal amplification for bacteria detection was utilized to amplify the signal and the results demonstrated proof of this concept that lectins, including WGA, can be utilized for signal amplification. With the optimized parameters, the proposed impedance immunosensor could detect *E. coli* O157:H7 at concentrations as low as 10^2^ cfu·mL^−1^with a linear detection range between 10^2^ and 10^7^ cfu·mL^−1^ (*r^2^* = 0.98) and a total detection time of less than 1 h. The cost of one SPIM can be further reduced by good reusability. Considering the ease of the electrodes and assay integration, the method we proposed presents an opportunity to develop a portable biosensing system for routine monitoring of foodborne pathogens.
